# Resolution of a Protracted Serogroup B Meningococcal Outbreak with Whole-Genome Sequencing Shows Interspecies Genetic Transfer

**DOI:** 10.1128/JCM.00881-16

**Published:** 2016-11-23

**Authors:** Robert M. Mulhall, Carina Brehony, Lois O'Connor, Kenneth Meyler, Keith A. Jolley, James Bray, Desiree Bennett, Martin C. J. Maiden, Robert Cunney

**Affiliations:** a Irish Meningitis and Sepsis Reference Laboratory, Temple Street Children's University Hospital, Dublin, Republic of Ireland; b Department of Zoology, University of Oxford, Oxford, England, United Kingdom; c Department of Public Health, HSE East, Dr Steevens' Hospital, Dublin, Republic of Ireland; University of Iowa College of Medicine

## Abstract

A carriage study was undertaken (*n* = 112) to ascertain the prevalence of Neisseria spp. following the eighth case of invasive meningococcal disease in young children (5 to 46 months) and members of a large extended indigenous ethnic minority Traveller family (*n* = 123), typically associated with high-occupancy living conditions. Nested multilocus sequence typing (MLST) was employed for case specimen extracts. Isolates were genome sequenced and then were assembled *de novo* and deposited into the Bacterial Isolate Genome Sequencing Database (BIGSdb). This facilitated an expanded MLST approach utilizing large numbers of loci for isolate characterization and discrimination. A rare sequence type, ST-6697, predominated in disease specimens and isolates that were carried (*n* = 8/14), persisting for at least 44 months, likely driven by the high population density of houses (*n* = 67/112) and trailers (*n* = 45/112). Carriage for Neisseria meningitidis (*P* < 0.05) and Neisseria lactamica (*P* < 0.002) (2-sided Fisher's exact test) was more likely in the smaller, more densely populated trailers. Meningococcal carriage was highest in 24- to 39-year-olds (45%, *n* = 9/20). Evidence of horizontal gene transfer (HGT) was observed in four individuals cocolonized by Neisseria lactamica and Neisseria meningitidis. One HGT event resulted in the acquisition of 26 consecutive N. lactamica alleles. This study demonstrates how housing density can drive meningococcal transmission and carriage, which likely facilitated the persistence of ST-6697 and prolonged the outbreak. Whole-genome MLST effectively distinguished between highly similar outbreak strain isolates, including those isolated from person-to-person transmission, and also highlighted how a few HGT events can distort the true phylogenetic relationship between highly similar clonal isolates.

## INTRODUCTION

Neisseria species, most of which reside in the nasopharynx, are considered harmless human commensal bacteria with two important exceptions: Neisseria gonorrhoeae, which inhabits the urogenital tract, and Neisseria meningitidis, a commensal bacterium which very rarely invades the nasopharyngeal epithelia and causes life-threatening septicemia and less frequently meningitis. In common with most countries worldwide, in the Republic of Ireland (RoI), invasive meningococcal disease (IMD) is legally notifiable (1). The RoI continues to experience an elevated rate of IMD relative to the rest of Europe despite the declining incidence observed over the last decade (1, 2). The majority of IMD is sporadic, with incidence in 2014 of 1.79/100,000 and 1.44/100,000 (80%) for serogroup B (3). As elsewhere, clusters are reported occasionally, typically in densely populated living environments such as military or university dormitories, with the protracted Princeton University outbreak, where the outbreak strain belonged to the hyperinvasive ST-41/44 clonal complex (cc), representing one such recent example from the United States (4).

Meningococcal populations are composed of multiple distinct lineages known as clonal complexes, according to the globally accepted bacterial typing paradigm, multilocus sequence typing (MLST) (5). Lineages consist of many highly similar strains, or sequence types (STs), which arise as a consequence of both clonal decent and the high rates of horizontal gene transfer (HGT) observed in meningococci (6, 45).

Meningococcal disease rates vary with age, with incidence rates peaking and declining within the first year of life until the mid to late teenage years when a second smaller incidence peak is observed (7), concomitant with the highest rates of carriage (8) and possibly a consequence of increased social activity. Many risk factors for IMD have been reported, such as complement cascade deficiencies, smoking, and socioeconomic status (9–11). The most important factor is the frequency and closeness of social contacts.

Endemic strain disease incidence can be controlled by vaccines targeting the polysaccharide that defines the meningococcal serogroup, with the important exception of serogroup B, which is poorly immunogenic in humans (12). Close contacts of individuals with IMD are typically offered chemoprophylaxis to reduce the occurrence of secondary cases. The National Guidelines for the Early Clinical and Public Health Management of Bacterial Meningitis define an outbreak of meningococcal disease as “a minimum of four cases of definite meningococcal disease within a 3-month interval, or 40/100,000 in any age group in a 3 month period, in a geographical area that makes epidemiological sense AND where available microbiological characterization of the organism is the same” (13).

Meningococcal outbreaks are less frequently observed than instances of endemic sporadic disease and tend to occur in environments where population densities are high, facilitating increases in both transmission rates and carriage and increasing the likelihood of persistent carriage.

Between March 2010 and November 2013, eight cases of IMD occurred in young children (5 to 46 months), who were members of an extended Irish Traveller family. Irish Travellers exhibit a unique culture and lifestyle (14), and as an indigenous minority, they experience social and cultural marginalization (15). No studies describing meningococcal disease outbreaks in a Traveller population have been reported previously. One month after the final case, a carriage study was undertaken to ascertain meningococcal prevalence. We present the molecular typing results of disease and carriage isolates, characterized by whole-genome sequencing (WGS), as an exemplar demonstrating how housing density can drive meningococcal carriage and facilitate persistence. In compliance with the National Guidelines and paralleling the Princeton experience, chemoprophylaxis was administered to close contacts following each case, but this failed to clear the outbreak strain from carriage in the larger network. Both outbreaks were controlled but only following mass chemoprophylaxis and simultaneous vaccination.

## MATERIALS AND METHODS

### Cohort.

This extended family lived in three separate midland towns, either in town housing or in trailers. Community members (*n* = 123) were invited to special outbreak control clinics; 112 choose to attend. The overall community size is somewhat larger (approximately 140 people) as some members were in other parts of Ireland or the UK and were unavailable to attend the clinics.

### Bacterial isolates and extracts.

The presence of N. meningitidis DNA was confirmed by real-time PCR (16) in specimen extracts from individuals with suspected IMD (*n* = 8, blood or cerebrospinal fluid [CSF]) and characterized using MLST (5, 17) and also PorA (18) and FetA (19) variable region (VR) typing. Two cases (B and H) yielded an N. meningitidis isolate which, together with the N. meningitidis isolates cultured from carriage (*n* = 14), was further characterized by serotyping (20), genogrouping (21), and multilocus restriction typing (MLRT) (22).

N. meningitidis carriage prevalence was evaluated by collecting anonymized pharyngeal swabs from 112/123 family members who attended special outbreak control clinics in December 2013. Specific demographic data were collected, including age, housing type, and housing occupancy. Pharyngeal swabs were cultured for Neisseria spp. The presence of meningococcal nucleic acid was investigated from swab head solutions using a *ctrA/porA* duplex real-time N. meningitidis-specific PCR assay (16).

### Whole-genome sequencing.

Genomic DNA was purified using the Wizard kit (Promega) according to the manufacturer's instructions. DNA quantity was assessed using the Qubit device (Life Technologies). DNA integrity was checked using 1% agarose gel (Promega). Genomic DNA from case and pharyngeal carriage isolates were sequenced on an Illumina HiSeq sequencer (Wellcome Trust Center for Human Genetics, University of Oxford, UK). The resulting short-read sequences were assembled *de novo* using the VelvetOptimiser algorithm (23) as part of an in-house pipeline developed in Oxford and then added to the publicly accessible Neisseria PubMLST website (24) (http://pubmlst.org/neisseria/), which uses the Bacterial Isolate Genome Sequence Database (BIGSdb) platform (24).

### Data analysis.

The BIGSdb Genome Comparator tool was used to investigate the relationships among the isolates. A specified set of loci among the isolates was compared, generating a matrix of allelic relatedness, which resolved isolates into a phylogenetic network using the Neighbor-Net algorithm (25) viewed in SplitsTree4 (26).

To verify that the assembled N. meningitidis sequences appearing to contain recombinant fragments from Neisseria lactamica were not due to contamination and/or misassembly, the short-read mapping programs Bowtie (27) and SRST2 (28) were used. R version 3.2.0 was used for both statistical analyses and the Venn diagram, using the “venn” function from the “gplots” package (www.r-project.org).

## RESULTS

### Molecular typing results of suspected IMD cases.

**FIG 1:**
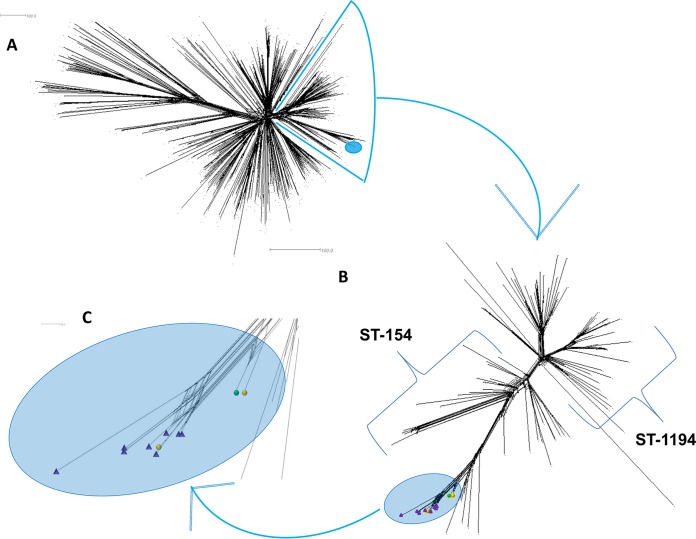
(A) Depiction of genetic relatedness based on a core genome allele by allele comparison of all ST-41/44 complex meningococci (*n* = 513) in the Meningitis Research Foundation Meningococcus Genome Library, representing all invasive isolates from the United Kingdom and Ireland. Outbreak isolates are highlighted in the light blue oval. (B) Individual cluster of 112 ST-154/ST-1194 meningococci, including variants. Highlighted are 11 ST-6697 strains (blue oval) including carried strains (*n* = 8, purple triangles) and invasive strains (*n* = 3, circles). An example of ST-6697 from the general population is indicated by the green circle, and cases B and H are represented by orange circles. Scale bars represent the counts of the observed differences at the 1,605 alleles compared. (C) Enlarged blue oval from panel (B) with 11 outbreak-related meningococcal highlighted as before.

Real-time PCR confirmed the presence of both meningococcal DNA and serogroup B capsular polysaccharide synthesis genes in all case-associated specimens received in the Irish Meningococcal and Meningitis Reference Laboratory (IMMRL). Case B and H isolates were identified as B:4:P1.7-2,4:ST-6697(cc41/44) with FetA VRs F1-21 and F5-12, respectively (Table 1). Case C and E isolates were identified as ST-6697(cc41/44). Incomplete MLST profiles were obtained for cases A (*aroE* and *pgm*) and F (*abcZ* and *pgm*) but were identical at the typed loci to those in the outbreak strain ST6697 (Table 1). All six strains shared the PorA VR type P1.7-2,4. A comparison of the ST6697 strains from this outbreak and the cc41/44 invasive isolates in the Meningitis Research Foundation Meningococcus Genome Library (MRF-MGL) revealed the rare nature of this ST, where all outbreak-related isolates clustered together and branched off a clade composed of ST154 or ST154 variants (Fig. 1).

**TABLE 1:** Associated epidemiological information and typing results for the eight outbreak cases

Characteristic	Results for case^*a*^:							
	A	B^*b*^	C	D	E	F	G	H^*b*^
Associated patient information								
Received date (day/mo/yr)	09/03/2010	22/11/2010	10/03/2011	06/01/2012	21/03/2013	30/04/2013	13/06/2013	28/11/2013
Age (mo)	6	5	5	10	15	46	9	6
Town	A	A	A	B	A	A	B	A
Lineage typing								
*abcZ*	3	3	3	4	3			3
*adk*	6	6	6	5	6	6		6
*aroE*		9	9	2	9	9		9
*fumC*	24	24	24		24	24		24
*gdh*	11	11	11		11			11
*pdhC*	6	6	6		6	6		6
*pgm*		9	9	20	9			9
MLST		6697	6697		6697			6697
Clonal complex	41/44	41/44	41/44		41/44	41/44		41/44
*rplF*		8						8
Antigen typing								
PorA type	P1.7-2,4	P1.7-2,4	P1.7-2,4		P1.7-2,4	P1.7-2,4		P1.7-2,4
FetA type	F1-21	F1-21	F5-12		F5-12			F5-12
fHbp peptide		4						4
NHBA peptide		607						Absent

a Cases B and H yielded an isolate on culture, while cases A, C, D, E, F, and G yielded CSF or blood extracts only.

b *NadA* was absent in both case B and H isolates.

### Carriage: age and dwelling types.

Pharyngeal swabs were obtained from 112 family members, yielding 14 cultures of N. meningitidis. One further N. meningitidis-positive carrier was identified by real-time PCR of swab head solutions, resulting in a carriage rate of 13.4% (*n* = 15/112) (29) (Table 2). N. lactamica was isolated from 31.25% (*n* = 35/112) of pharyngeal swabs, and Neisseria mucosa was isolated once (0.9%). Both N. meningitidis and N. lactamica were isolated from 3.6% (*n* = 4/112) of individuals. The age distribution of this family was notable in that 66% were younger than 20 years of age (52.2% for the general Traveller population [*n* = 29,573] and 27.2% for the general Irish population [2011 census data]).

**TABLE 2:** Associated epidemiological information and typing results for meningococcus-positive pharyngeal swabs (*n* = 15/112)

Characteristic	Results for laboratory swab number:														
	M1	M2	M13	M14	M16	M20	M30	M31	M32	M34	M35	M42	M46	M45	Unassigned^*a*^
Associated carrier information															
Age (yr)	28	34	16	35	15	38	18	32	32	27	25	38	3	6	45
Town	A	A	A	A	B	C	C	C	C	C	C	C	C	C	B
Housing type^*b*^	Trailer	Trailer	House	Trailer	House	House	Trailer	Trailer	Trailer	Trailer	Trailer	House	Trailer	Trailer	House
Lineage															
MLRT	36	36	36	6	219	121	12	36	219	36	36	8	36	36	
rMLST^*c*^	2560	2560	2560	ND^*d*^	ND	ND	ND	2560	ND	2560	2560	ND	17376	ND	
Sequence type	6697	6697	6697	7244	10659	1163	1281	6697	10659	6697	6697	1157	6697	6697	
Clonal complex	41/44	41/44	41/44	461		269	22	41/44		41/44	41/44	1157	41/44	41/44	
Serogroup and antigen data															
Serogroup	B	B	B	B	B	B	W	B	B	B	B	NG	B	B	B
PorA type	P1.7-2,4	P1.7-2,4	P1.7-2,4	P1.19-2,13-1	P1.22, 9	P1.22, 9	P1.18-1, 3	P1.7-2,4	P1.22, 9	P1.7-2,4	P1.7-2,4	P1.21-7, 16	P1.7-2,4	P1.7-2,4	
FetA type	F5-12	F5-12	F5-12	F3-9	F1-7	F1-7	F1-2	F5-12	F1-7	F5-12	F5-12	F5-36	F5-43	F5-43	
fHbp peptide	4	4	4	47	13	19	16	4	13	4	4	13	4	4	
NHBA peptide	607	607	607	118	17	17	20	-	17	-	607	114	607	607	

a Swab head solution tested positive for meningococcal nucleic acid by real-time PCR, no isolated was cultured, and therefore no M number assigned.

b Individual family members lived in trailers (*n* = 45/112) and in houses (*n* = 67/112).

c rMLST, ribosomal multilocus sequence typing.

d ND, not determined.

**FIG 2:**
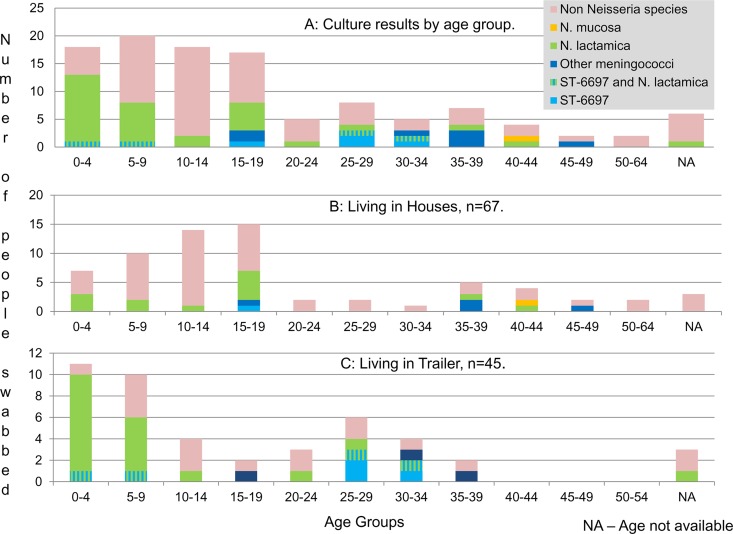
Culture results of 112 swabs volunteered by extended Traveller family members by age group (A) and by age group and housing types, house (B) and trailer (C).

The majority of N. meningitidis isolates (*n* = 10/15) were obtained from adults between 25 and 39 years of age (45% carriage, *n* = 9/20), 3 were from 14- to 19-year-olds (17.6% carriage, *n* = 3/17), and 2 were from children younger than 9 years of age (5.3% carriage, *n* = 2/38) (Fig. 2). N. lactamica isolates (*n* = 35) were obtained mostly from 0- to 9-year-olds (55.3% carriage, *n* = 21/38), 5 were from 15- to 19-year-olds (29.4% carriage, *n* = 5/17), and 5 were from those 25 years and older (25% carriage, *n* = 5/25) (Fig. 2).

The average occupancy for those living in houses (*n* = 67) was 6.7 people with carriage rates of 7.5% (*n* = 5/67) and 19.4% (*n* = 13/67) observed for N. meningitidis and N. lactamica, respectively. Higher carriage rates of 22.2% (*n* = 10/45) for N. meningitidis and 48.9% (*n* = 22/45) for N. lactamica were observed in trailers (*n* = 45), where the average occupancy was 7.2 people per dwelling. An overall effect of housing type for the carriage of N. meningitidis (*P* < 0.05) and N. lactamica (*P* < 0.002) (2-sided Fisher's exact test) was observed. The effect of housing type and age-specific carriage was also investigated for both species (see Table S1 at http://imsrl.zohosites.com/files/Suplemental_Materials_MulhallRM_JCM00881-16.pdf). In general, carriage was more likely in trailers than in houses. Dwelling density as a factor for carriage was also explored. Those dwelling in homes with ≥8 people were more likely to carry N. lactamica (odds ratio [OR], 3.79; 95% confidence interval [CI_95_], 1.44 to 9.94; *P* = 0.005), while a similar but weaker effect was observed for N. meningitidis (OR, 3.26; CI_95_, 0.84 to 12.63; *P* = 0.067). Age-specific dwelling density was explored for both N. meningitidis and N. lactamica, where generally carriage was more likely for those in a dwelling with ≥8 occupants (see Table S2 at http://imsrl.zohosites.com/files/Suplemental_Materials_MulhallRM_JCM00881-16.pdf). Higher average bedroom occupancies were observed for trailers (*n* = 4.49 people) than for houses (*n* = 1.95 people). As bedroom occupancy increased, carriage rates for both N. meningitidis and N. lactamica also increased (see Table S3 at http://imsrl.zohosites.com/files/Suplemental_Materials_MulhallRM_JCM00881-16.pdf).

The high meningococcal carriage (45%, *n* = 9/20) in the 25- to 39-year-olds may be a consequence of the especially crowded living conditions experienced by several individuals in this age group. Positive carriers (*n* = 9) and negative carriers (*n* = 11) had average occupancies per dwelling of 8.1 people and 5.2 people, respectively, and average per bedroom occupancies of 4.7 people and 2.1 people, respectively.

Although a likely confounding factor, smoking could not be considered because data on smoking were not collected. It should be noted that carriage studies are point estimates of carriage and may or may not be representative of carriage rates over longer periods of time. Care must be exercised when inferring from the associated data collected, as living arrangements may also be subject to variations over time. Further, several of the cohort had been offered chemoprophylaxis in the previous 4 months (*n* = 39/112).

### Meningococcal sequence types carried.

**FIG 3:**
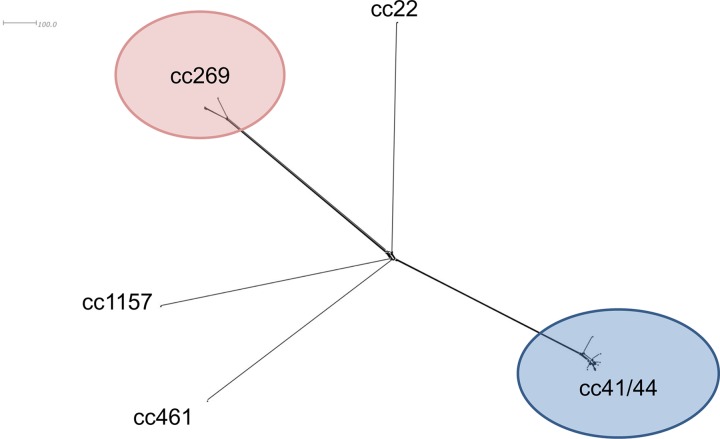
Neighbor-Net diagram of the 14 isolates recovered from nasopharyngeal swabs, generated from 1,605 core meningococcal genes. Included are eight ST-6697 isolates (cc41/44), three cc269 isolates, and one isolate each from cc22, cc461, and cc1157.

The 14 N. meningitidis isolates cultured that were carried included 12 serogroup B isolates; 8 were identified as B:P1.7-2,4:ST-6697(cc41/44), the outbreak strain. Five clonal complexes were represented among the isolates carried (Fig. 3). Five of the eight ST6697 isolates were from individuals aged 24 to 34 years. Seven ST6697 isolates were recovered from individuals living in trailers (*n* = 7/45).

### Comparison of ST6697 isolates.

**FIG 4:**
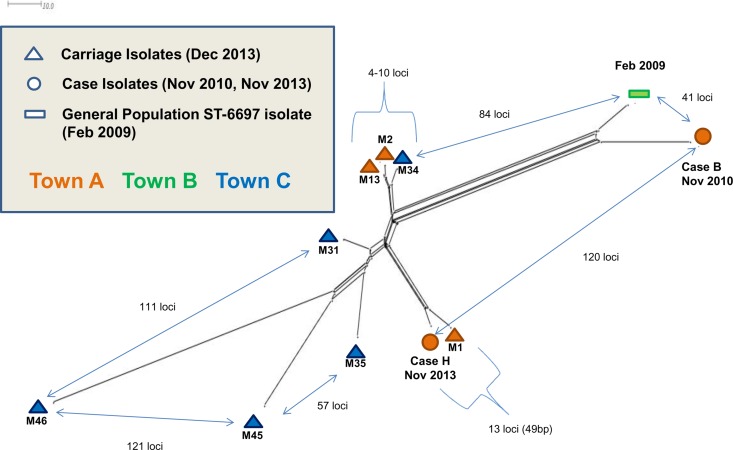
Neighbor-Net diagram depicting genetic relationships of ST-6697 isolates generated from an allele-by-allele comparison of 1,505 core meningococcal genes. Carried strains recovered from throat swabs volunteered by the family members in December 2013 (*n* = 8, triangles) and case isolates B and H (circles). Also included is a single example of an ST-6697 meningococcus from a historical IMD case with an address in one of the towns where the family members reside (town B), isolated 13 months prior to the start of the outbreak (green bar).

The relatedness of 11 ST6697 isolates, based on an allele-by-allele comparison of the core N. meningitidis genome (1,605 loci), was determined (Fig. 4). This included eight carriage isolates (December 2013), outbreak case B and H isolates (November 2010 and 2013, respectively), and one ST6697 isolate from an individual from the general population living in town B and predating the outbreak (February 2009). The latter isolate differed from the case B isolate at 41/1,605 loci, and both isolates were more related to each other than to the other meningococcal isolates. Carriage isolate M1 showed the greatest similarity to the most recent invasive isolate, case H, differing at 13/1,605 core loci (0.8%), representing 49 nucleotide differences. This very likely represents direct transmission among family members of the same strain that caused invasive disease 1 month earlier. Isolates M2, M13, and M34 represent three variants of another meroclone, grouped very closely to each other but not to any other isolates, again representing direct person-to-person transmission.

### Case B and H isolate comparison.

Whole-genome MLST comparison of isolates from case B (March 2010) and case H (November 2013) revealed that 8.5% of alleles were dissimilar (163/1,887), with 71.8% (117/163) of this allelic variation found in the core genome. Eight regions that had five or more consecutive variant alleles were identified, suggesting that these alleles were imported by HGT. Many of these alleles encoded gene products with known or presumptive metabolic functionality. The largest region contained 26 loci (NEIS2124 to NEIS2153) and, on the basis of sequence comparison, was likely imported from cc162 meningococci (data not shown).

### Interspecies recombination.

N. meningitidis and N. lactamica were cocultured from pharyngeal swabs on four occasions. Pharyngeal swabs M31, M35, M45, and M46 were volunteered by individuals dwelling in trailers, who were aged 32, 25, 6, and 3, and years, respectively. ST6697 isolates from pharyngeal swabs M45 and M46 were comparatively dissimilar to the other contemporaneously isolated meningococcal ST6697 strains as a result of cocolonization and HGT with N. lactamica (Fig. 4).

**FIG 5:**
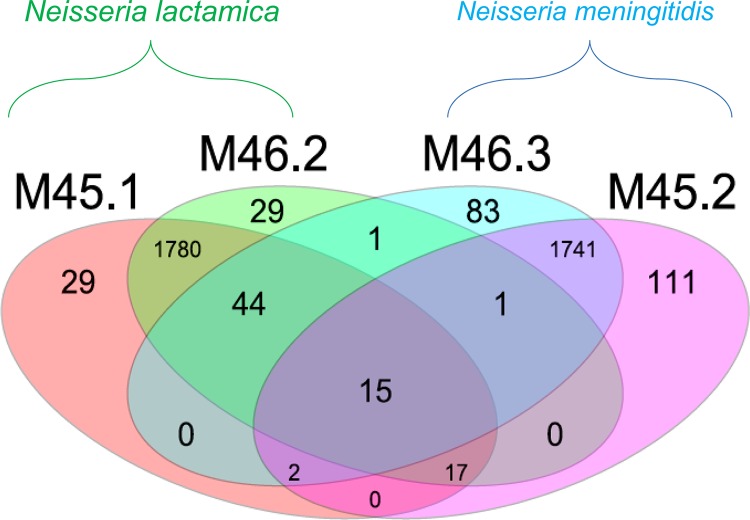
Venn diagram of a whole-genome MLST comparison (loci, 1,887) showing shared alleles for N. meningitidis (M46.3 and M45.2) and N. lactamica (M45.1 and M46.2) isolates from nasopharyngeal carriage in two individuals. Cocolonizing isolates M45.1 and M45.2 shared 34 alleles while cocolonizing isolates M46.3 and M46.2 shared 61 alleles.

A whole-genome comparison (1,887 loci) of two N. lactamica isolates (ST10984) and two meningococcal isolates (ST6697) cultured from their respective pharyngeal swabs was undertaken (Fig. 5). The two N. lactamica isolates shared alleles at 1,856 of 1,887 loci (98.36%). Similarly, the meningococcal isolates shared alleles at 1,759 of 1,887 loci (93.2%). All four isolates shared identical alleles at 15 loci. Meningococcal isolate M45.2 contained 34 and 33 alleles that were identical to those in N. lactamica isolates M45.1 and M46.2, respectively. Meningococcal isolate M46.3 shared 61 identical alleles each with N. lactamica isolates M45.1 and M46.2. For comparison, the next most closely related ST6697 isolate to M46.2, the carried isolate M35.1, shared seven alleles with it. Shared alleles among M45.1, M45.2, M46.2, and M46.3 included the iron acquisition gene, *fetA* (F5-43), a transferrin binding protein, *tbpB*, and a prepilin peptidase, *pilD*. Many of these shared alleles were observed adjacent to each other in the meningococcal genomes, suggesting acquisition via HGT. Short-read mapping-based sequence assembly tools Bowtie and SRST2 verified that neither contamination nor misassembly was the cause of this higher than usual sharing of alleles among the species.

Nineteen N. lactamica isolates were genome sequenced; 16 of these were identified as ST10984 and shared between three and five identical alleles with both the invasive ST6697 isolates and the four carried meningococcal ST6697 isolates not found cocolonizing with N. lactamica. All four cocolonizing ST6697 isolates contained a greater number of shared alleles (see Fig. S1 at http://imsrl.zohosites.com/files/Suplemental_Materials_MulhallRM_JCM00881-16.pdf). Together these isolates likely represent donor and recipient strains before and after HGT.

## DISCUSSION

Although rare in many settings, meningococcal serogroup B outbreaks can be both protracted in nature and highly variable in scale, some prompting the vaccination of millions of individuals, for example, the experiences in New Zealand (30), Chile (31), Cuba (32), and Norway (33). Outbreaks have been described in institutional settings, and these too can be protracted; for example, an outbreak in a crèche in Warwickshire, UK, lasted 4 months and was caused by B:ST-1194(cc41/44) meningococci (34). Other high-risk settings include military camps and universities, exemplified by the recent outbreak at Princeton, where the outbreak strain (B:ST-409:cc41/44) persisted for 12 months and spanned two academic years. In the current outbreak, the ST-6697 outbreak strain persisted in the Traveller cohort for at least 44 months, and 1 month after the final case, significantly higher carriage rates of N. meningitidis and N. lactamica were observed in the inhabitants of the trailers, which were more densely populated. This is consistent with many observations made since Glover described a meningococcal epidemic in 1917 at “X” Depot where, as the number of cases increased over the winter months, carriage rates increased dramatically (35). After the number of military personal beds per barrack room was decreased, the epidemic was much less severe 1 year later as this intervention impacted the carriage of and disease caused by the meningococcus (35).

In the Traveller cohort investigated here, the high carriage rates presumably facilitated persistence of the outbreak strain and subsequent transmission to infants and young children. The high compliance for chemoprophylaxis and vaccination (Bexsero) (36) probably cleared the strain, as no new cases have occurred since November 2013. It is known that chemoprophylaxis can prevent secondary cases, but controlling such outbreaks effectively requires mass chemoprophylaxis and simultaneous vaccination to protect individuals should chemoprophylaxis fail to purge the strain from the larger network. Before these measures can be implemented, the outbreak must first be recognized, which ideally requires real-time high-resolution characterization of IMD case isolates and a rapid and reproducible way of relating isolates to each other and to endemic disease strains. Specific national recommendations for high-risk groups might also be conducive to early outbreak recognition.

Conventional MLST, augmented by antigen typing (*porA* and *fetA*), has sufficient discriminatory resolution for both national and international epidemiological analysis and has been routinely implemented (37). However, isolates from outbreaks are typically indistinguishable by this approach, given the short periods of time involved to accumulate variation, and so this scheme is less useful for understanding the epidemiology of very closely related isolates. The application of WGS data allows the expansion of the MLST principle (38) to include more genes, for example, the core meningococcal gene scheme (*n* = 1,605) (39). This provides high discrimination and greater robustness for epidemiological analysis. The present study illustrates the capacity of core genome MLST (cgMLST) to unambiguously resolve a group of extremely similar outbreak strain isolates, including those recovered from person-to-person transmission. Consequently, whole-genome MLST may be applied to investigate the transmission dynamics of pathogenic bacteria in human cohorts. For example, the elucidation of transmission chains among permanent hospital staff may provide an opportunity for intervention, by revealing how staff or their working environments facilitate the persistence of antimicrobial-resistant strains and their subsequent transmission to patients.

Horizontal gene transfer (HGT) is common among meningococci, and its complicating effects on strain characterization have long been recognized (6, 39). Evidence of inter- and intraspecies HGT can be observed by the differential allele frequencies that exist within and among different Neisseria species (http://pubmlst.org/neisseria/). In addition, shared nucleotide sequence polymorphisms are present in numerous loci, including those encoding certain common antigenic loci such as the typing target FetA (40). Here we observed inter- and intraspecies HGT, including extensive introgression from an N. lactamica strain to a meningococcal strain present in the same individual. This is, to our knowledge, the first *in vivo* example of interspecies recombination and demonstrates how extensive HGT can be. At least 3% of the nucleotide content of the introgressed meningococcus isolate M46.3 had been replaced with N. lactamica DNA, including a large single event which contained 26 newly acquired alleles. This demonstrates that care is required in the investigation of outbreaks caused by highly recombinogenic organisms, as many genetic differences can be introduced rapidly by a few HGT events. Understanding the nature of genetic variation among such isolates is therefore important. Alleles can vary by a single nucleotide, which is likely the consequence of point mutation, whereas multiple nucleotide differences are likely the consequence of HGT with a different strain or species. Allelic variation may occur from nucleotide insertions/deletions resulting from HGT with sister cells, which was commonly observed among the ST6697 carried isolates that were studied here.

The study further demonstrated the effect of housing density on the carriage of N. meningitidis and N. lactamica and the way that high carriage facilitated strain persistence. It also emphasized the utility of WGS to augment conventional MLST typing and unambiguously discriminate extremely closely related isolates and illustrated the potential effectiveness of cgMLST as a novel public health tool for early outbreak detection. As this level of strain characterization becomes routine, further examples of HGT among Neisseria species will emerge. Understanding the dynamics of the antigenic surface structure exchange between Neisseria species has potential consequences for vaccine design and postimplementation monitoring, especially since the relationships between commensal bacteria and pathogenic species are poorly understood. N. lactamica carriage peaks in the first years of life when it is inversely correlated with low N. meningitidis carriage (41). Carriage of N. lactamica has also been demonstrated to confer protection against the development of IMD (42). Currently licensed meningococcal serogroup B substitute vaccines, the four-component vaccine Bexsero (36) and the bivalent Trumenba (43), utilize antigens which exhibit evidence of frequent interspecies HGT with commensal Neisseria (44), with the exception of the PorA component (Bexsero) which is unique among nasopharyngeal Neisseria species to the meningococcus. Neisserial heparin-binding antigen (Bexsero) is common to several Neisseria species, including N. lactamica (44).

In conclusion, this study shows how a single meningococcal strain with relatively high invasive potential can establish itself in a semiclosed population, causing a persistent disease outbreak. Although in this case the outbreak was very likely exacerbated by the particular setting, including individuals housed in trailers and the consequent close proximity of people of different age groups, this is very likely similar to what happens in larger population and on longer time scales, such as the Stonehouse outbreak in the UK in the 1980s and the New Zealand outbreak in the 1990s. The diversity of the outbreak strain over 4 years in spreading to different towns was limited, with the greatest degree of variation occurring by a limited number of HGT events, which resulted in the introduction of multiple variant alleles.

## References

[1] Health Protection Surveillance Centre. 2011 Computerised infectious disease reporting—CIDR. https://www.hpsc.ie/CIDR/Presentations/File,1112,en.pdf.

[2] Trotter CL, Chandra M, Cano R, Larrauri A, Ramsay ME, Brehony C, Jolley KA, Maiden MCJ, Heuberger S, Frosch M 2007 A surveillance network for meningococcal disease in Europe. FEMS Microbiol Rev 31:27–36. doi:10.1111/j.1574-6976.2006.00060.x. 17168995

[3] Health Protection Surveillance Centre. 2014 Annual report 2014. Health Protection Surveillance Centre, Dublin, Ireland http://www.hpsc.ie/AboutHPSC/AnnualReports/File,15505,en.pdf.

[4] McNamara LA, Shumate AM, Johnsen P, MacNeil JR, Patel M, Bhavsar T, Cohn AC, Dinitz-Sklar J, Duffy J, Finnie J, Garon D, Hary R, Hu F, Kamiya H, Kim HJ, Kolligian J Jr, Neglia J, Oakley J, Wagner J, Wagner K, Wang X, Yu Y, Montana B, Tan C, Izzo R, Clark TA 2015 First use of a serogroup B meningococcal vaccine in the US in response to a university outbreak. Pediatrics 135:798–804. doi:10.1542/peds.2014-4015. 25917990 PMC4620546

[5] Maiden MC, Bygraves JA, Feil E, Morelli G, Russell JE, Urwin R, Zhang Q, Zhou J, Zurth K, Caugant DA, Feavers IM, Achtman M, Spratt BG 1998 Multilocus sequence typing: a portable approach to the identification of clones within populations of pathogenic microorganisms. Proc Natl Acad Sci U S A 95:3140–3145. doi:10.1073/pnas.95.6.3140. 9501229 PMC19708

[6] Feavers IM, Heath AB, Bygraves JA, Maiden MCJ 1992 Role of horizontal genetic exchange in the antigenic variation of the class 1 outer membrane protein of Neisseria meningitidis. Mol Microbiol 6:489–495. doi:10.1111/j.1365-2958.1992.tb01493.x. 1560777

[7] Caugant DA, Maiden MCJ 2009 Meningococcal carriage and disease—population biology and evolution. Vaccine 27:B64−B70. doi:10.1016/j.vaccine.2009.04.061. 19464092 PMC2719693

[8] Christensen H, May M, Bowen L, Hickman M, Trotter CL 2010 Meningococcal carriage by age: a systematic review and meta-analysis. Lancet Infect Dis 10:853–861. doi:10.1016/S1473-3099(10)70251-6. 21075057

[9] Heyderman RS, Ben-Shlomo Y, Brennan CA, Somerset M 2004 The incidence and mortality for meningococcal disease associated with area deprivation: an ecological study of hospital episode statistics. Arch Dis Child 89:1064–1068. doi:10.1136/adc.2003.036004. 15499066 PMC1719724

[10] Kriz P, Bobak M, Kriz B 2000 Parental smoking, socioeconomic factors, and risk of invasive meningococcal disease in children: a population based case-control study. Arch Dis Child 83:117–121. doi:10.1136/adc.83.2.117. 10906015 PMC1718425

[11] Davila S, Wright VJ, Khor CC, Sim KS, Binder A, Breunis WB, Inwald D, Nadel S, Betts H, Carrol ED, de Groot R, Hermans PWM, Hazelzet J, Emonts M, Lim CC, Kuijpers TW, Martinon-Torres F, Salas A, Zenz W, Levin M, Hibberd ML 2010 Genome-wide association study identifies variants in the CFH region associated with host susceptibility to meningococcal disease. Nat Genet 42:772–776. doi:10.1038/ng.640. 20694013

[12] Finne J, Leinonen M, Mäkelä PH 1983 Antigenic similarities between brain components and bacteria causing meningitis. Implications for vaccine development and pathogenesis. Lancet ii:355–357. 10.1016/s0140-6736(83)90340-9 6135869

[13] Bacterial Meningitis Sub-Committee of the Scientific Advisory Committee of the HPSC. 2012 Guidelines for the early clinical and public health management of bacterial meningitis (including meningococcal disease). Health Protection Surveillance Centre, Dublin, Ireland.

[14] All Ireland Traveller Health Study Team. 2010 All Ireland Traveller Health Study: our geels. Summary of findings. Minister for Health and Children Mary Harney, Dublin, Ireland.

[15] Stephens C, Porter J, Nettleton C, Willis R 2006 Disappearing, displaced, and undervalued: a call to action for indigenous health worldwide. Lancet 367:2019–2028. doi:10.1016/S0140-6736(06)68892-2. 16782493

[16] Borrow R, Claus H, Guiver M, Smart L, Jones DM, Kaczmarski EB, Frosch M, Fox AJ 1997 Non-culture diagnosis and serogroup determination of meningococcal B and C infection by a sialyltransferase (siaD) PCR ELISA. Epidemiol Infect 118:111–117. doi:10.1017/S0950268896007261. 9129587 PMC2808779

[17] Birtles A, Hardy K, Gray S 2005 Multilocus sequence typing of Neisseria meningitidis directly from clinical samples and application of the method to the investigation of meningococcal disease case. J Clin Microbiol 43:6007–6014. doi:10.1128/JCM.43.12.6007-6014.2005. 16333090 PMC1317198

[18] Russell JE, Jolley KA, Feavers IM, Maiden MCJ, Suker J 2004 PorA variable regions of Neisseria meningitidis. Emerg Infect Dis 10:674–678. doi:10.3201/eid1004.030247. 15200858 PMC3323080

[19] Thompson EAL, Feavers IM, Maiden MCJ 2003 Antigenic diversity of meningococcal enterobactin receptor FetA, a vaccine component. Microbiology 149:1849–1858. doi:10.1099/mic.0.26131-0. 12855736

[20] Wedege E, Hoiby EA, Rosenqvist E, Froholm LO 1990 Serotyping and subtyping of Neisseria meningitidis isolates by co-agglutination, dot-blotting and ELISA. J Med Microbiol 31:195–201. doi:10.1099/00222615-31-3-195. 2107317

[21] Bennett DE, Cafferkey MT 2006 Consecutive use of two multiplex PCR-based assays for simultaneous identification and determination of capsular status of nine common Neisseria meningitidis serogroups associated with invasive disease. J Clin Microbiol 44:1127–1131. doi:10.1128/JCM.44.3.1127-1131.2006. 16517911 PMC1393079

[22] Bennett DE, Cafferkey MT 2003 Multilocus restriction typing: a tool for Neisseria meningitidis strain discrimination. J Med Microbiol 52:781–787. doi:10.1099/jmm.0.05225-0. 12909655

[23] Zerbino DR 2010. Using the Velvet de novo assembler for short-read sequencing technologies. Curr Protoc Bioinformatics Chapter 11:Unit 11.5. 10.1002/0471250953.bi1105s31 PMC2952100 20836074

[24] Jolley KA, Maiden MCJ 2010 BIGSdb: scalable analysis of bacterial genome variation at the population level. BMC Bioinformatics 11:595. doi:10.1186/1471-2105-11-595. 21143983 PMC3004885

[25] Bryant D, Moulton V 2004 Neighbor-Net: an agglomerative method for the construction of phylogenetic networks. Mol Biol Evol 21:255–265. doi:10.1093/molbev/msh018. 14660700

[26] Huson DH, Bryant D 2006 Application of phylogenetic networks in evolutionary studies. Mol Biol Evol 23:254–267. doi:10.1093/molbev/msj030. 16221896

[27] Langmead B 2010 Aligning short sequencing reads with Bowtie. Curr Protoc Bioinformatics Chapter 11:Unit 11.7. 10.1002/0471250953.bi1107s32 PMC3010897 21154709

[28] Inouye M, Dashnow H, Raven L, Schultz MB, Pope BJ, Tomita T, Zobel J, Holt KE 2014 SRST2: rapid genomic surveillance for public health and hospital microbiology labs. Genome Med 6:90. doi:10.1186/s13073-014-0090-6. 25422674 PMC4237778

[29] O'Connor L, Ward M, Bennett D, Mulhall R, Lorcain PO, Cunney R, Mcdermott R, Neville E 2015 A prolonged outbreak of invasive meningococcal disease in an extended Irish Traveller family across three Health Service Executive (HSE) areas in Ireland, 2010 to 2013. Euro Surveill. 20(21):pii=21139 http://www.eurosurveillance.org/ViewArticle.aspx?ArticleId=21139. 10.2807/1560-7917.es2015.20.21.21139 26062560

[30] Oster P, Lennon D, O'Hallahan J, Mulholland K, Reid S, Martin D 2005 MeNZB: a safe and highly immunogenic tailor-made vaccine against the New Zealand Neisseria meningitidis serogroup B disease epidemic strain. Vaccine 23:2191–2196. doi:10.1016/j.vaccine.2005.01.063. 15755593

[31] Boslego J, Garcia J, Cruz C, Zollinger W, Brandt B, Ruiz S, Martinez M, Arthur J, Underwood P, Silva W, Moran E, Hankins W, Gilly J, Mays J 1995 Efficacy, safety, and immunogenicity of a meningococcal group B (15:P1.3) outer membrane protein vaccine in Iquique, Chile. Vaccine 13:821–829. doi:10.1016/0264-410X(94)00037-N. 7483804

[32] Sierra G, Campa HC, Varcacel NM, Garcia IL, Izquierdo PL, Sotolongo PF, Casanueva G, Rico CO, Rodriguez CR, Terry MH 1991 Vaccine against group B Neisseria meningitidis: protection trial and mass vaccination results in Cuba. NIPH Ann 14:195–210. 1812432

[33] Bjune G, Hoiby EA, Gronnesby JK, Arnesen O, Fredriksen JH, Halstensen A, Holten E, Lindbak A, Nøkleby H, Rosenqvist E 1991 Effect of outer membrane vesicle vaccine against serogroup B meningococcal disease in Norway. Lancet 338:1093–1096. doi:10.1016/0140-6736(91)91961-S. 1682541

[34] Chatt C, Gajraj R, Hawker J, Neal K, Tahir M, Lawrence M, Gray SJ, Lucidarme J, Carr AD, Clark SA, Fowler T 2014 Four-month outbreak of invasive meningococcal disease caused by a rare serogroup B strain, identified through the use of molecular PorA subtyping, England, 2013. Euro Surveill 19(44):pii=20949 http://www.eurosurveillance.org/ViewArticle.aspx?ArticleId=21139. 10.2807/1560-7917.es2014.19.44.20949 25394258

[35] Glover JA 1918 The cerebro-spinal fever epidemic of 1917 at “X” depot. J R Army Med Corps 30:23−36. 10.1017/s002217240000718x PMC2207078 20474675

[36] Bambini S, Piet J, Muzzi A, Keijzers W, Comandi S, De Tora L, Pizza M, Rappuoli R, van de Beek D, van der Ende A, Comanducci M 2013 An analysis of the sequence variability of meningococcal fHbp, NadA and NHBA over a 50-year period in the Netherlands. PLoS One 8:e65043. doi:10.1371/journal.pone.0065043. 23717687 PMC3663754

[37] Brehony C, Trotter CL, Ramsay ME, Chandra M, Jolley KA, Van Der Ende A, Carion F, Berthelsen L, Hoffmann S, Haroardóttir H, Vazquez JA, Murphy K, Toropainen M, Caniça M, Ferreira E, Diggle M, Edwards GF, Taha MK, Stefanelli P, Kriz P, Gray SJ, Fox AJ, Jacobsson S, Claus H, Vogel U, Tzanakaki G, Heuberger S, Caugant DA, Frosch M, Maiden MCJ 2014 Implications of differential age distribution of disease-associated meningococcal lineages for vaccine development. Clin Vaccine Immunol 21:847–853. doi:10.1128/CVI.00133-14. 24695776 PMC4054250

[38] Maiden MCJ, Jansen van Rensburg MJ, Bray JE, Earle SG, Ford SA, Jolley KA, Mccarthy ND 2013 MLST revisited: the gene-by-gene approach to bacterial genomics. Nat Rev Microbiol 11:728–736. doi:10.1038/nrmicro3093. 23979428 PMC3980634

[39] Bratcher HB, Corton C, Jolley KA, Parkhill J, Maiden MCJ 2014 A gene-by-gene population genomics platform: de novo assembly, annotation and genealogical analysis of 108 representative Neisseria meningitidis genomes. BMC Genomics 15:1138. doi:10.1186/1471-2164-15-1138. 25523208 PMC4377854

[40] Bennett JS, Thompson EAL, Kriz P, Jolley KA, Martin CJ 2009 A common gene pool for the Neisseria FetA antigen. Int J Med Microbiol 299:133–139. doi:10.1016/j.ijmm.2008.06.010. 18718812 PMC3968273

[41] Gold R, Goldschneider I, Lepow ML, Draper TF, Randolph M 1978 Carriage of Neisseria meningitidis and Neisseria lactamica in infants and children. J Infect Dis 137:112–121. doi:10.1093/infdis/137.2.112. 415097

[42] Evans CM, Pratt CB, Matheson M, Vaughan TE, Findlow J, Borrow R, Gorringe AR, Read RC 2011 Nasopharyngeal colonization by Neisseria lactamica and induction of protective immunity against Neisseria meningitidis. Clin Infect Dis 52:70–77. doi:10.1093/cid/ciq065. 21148522

[43] Nissen M, Marshall H, Richmond P, Jiang Q, Harris S, Jones T, Jansen K, Perez J 2013 A randomized, controlled, phase 1/2 trial of a Neisseria meningitidis serogroup B bivalent rLP2086 vaccine in healthy children and adolescents. Pediatr Infect Dis 32:364–371. doi:10.1097/INF.0b013e31827b0d24. 23114369

[44] Muzzi A, Mora M, Pizza M, Rappuoli R, Donati C 2013 Conservation of meningococcal antigens in the genus Neisseria. mBio 4(3):e00163–13. 23760461 10.1128/mBio.00163-13 PMC3685207

[45] Holmes EC, Urwin R, Maiden MCJ 1999 The influence of recombination on the population structure and evolution of the human pathogen Neisseria meningitidis. Mol Biol Evol 16:741–749. doi:10.1093/oxfordjournals.molbev.a026159. 10368953

